# Pandemic-related socioeconomic disruptions and adverse health outcomes: a cross-sectional study of female caregivers

**DOI:** 10.1186/s12889-022-14287-2

**Published:** 2022-10-11

**Authors:** Erika M. Brown, Lia C.H. Fernald, Rita Hamad, Mekhala Hoskote, Kaitlyn E. Jackson, Wendi Gosliner

**Affiliations:** 1 Social Interventions Research and Evaluation Network, Center for Health and Community, University of California, San Francisco, 675 18th St, 94107 San Francisco, CA USA; 2 Division of Community Health Sciences, School of Public Health, University of California, Berkeley, 2121 Berkeley Way, Room 5302, 94720 Berkeley, CA USA; 3 Philip R. Lee Institute for Health Policy Studies, Department of Family & Community Medicine, University of California, 490 Illinois St, 94158 San Francisco, CA USA; 4 UC Berkeley-UCSF Joint Medical Program, 570 University Hall MC #7360, 2018 Oxford Street, 94720 Berkeley, CA USA; 5 Philip R. Lee Institute for Health Policy Studies, University of California, 490 Illinois St, 94158 San Francisco, CA USA; 6 Nutrition Policy Institute, Division of Agriculture and Natural Resources, University of California, 1111 Franklin Street, 94607 Oakland, CA USA

**Keywords:** Parental health, Socioeconomic factors, COVID-19, Mental health, Food insecurity, Child care, Employment, Housing

## Abstract

**Background:**

The COVID-19 pandemic and efforts to mitigate transmission resulted in sudden and widespread socioeconomic disruptions including school and child care closures, unemployment and underemployment, and housing precarity. Understanding the extent to which these disruptions may have contributed to adverse health outcomes is critical for establishing policy priorities that can mitigate further harm.

**Methods:**

We explored the associations between pandemic-related child care, employment, and housing disruptions with depressive symptoms, self-rated health, and food security status among a sample of economically disadvantaged and racially diverse female caregivers of young children (n=464). Data were derived from the Assessing California Communities’ Experiences with Safety Net Supports (ACCESS) study, which conducted survey-based interviews with California caregivers with low-income from August 2020 – May 2021. We implemented a series of multivariable Poisson regressions with robust standard errors to assess the potency of each exposure, independently and within the context of one another.

**Results:**

Most caregivers experienced disruptions to child care (70%) and employment (63%); few experienced major housing disruptions (8%). Women that experienced child care and housing disruptions had greater depressive symptoms, lower self-rated health, and greater food insecurity, although the relationships for housing and depressive symptoms were modified by the timing of participants’ interviews. Employment disruptions were not associated with any of the examined adverse health outcomes.

**Conclusion:**

In the wake of socioeconomic stressors brought about by the COVID-19 pandemic, attending to structural deficits in the child care system and increasing housing supports may be critical for protecting the health of caregivers.

**Supplementary information:**

The online version contains supplementary material available at 10.1186/s12889-022-14287-2.

## Background

The COVID-19 pandemic has challenged communities in the United States (US) and across the globe, ushering in widespread morbidity, excess mortality, and mass disruptions to daily life.[1, 2] Efforts to stymie transmission resulted in sudden and widespread un- and underemployment, school and child care closures and reduced availability of health and certain social services.[3–5] While US residents collectively endured this sudden societal upheaval, its impacts have been unequally distributed.[5–9] Pandemic disruptions have compounded issues arising from longstanding racial and socioeconomic inequities, early child care and education disparities, and the country’s inadequate supply of affordable housing, disproportionately impacting marginalized families and female caregivers, in particular.[7–15]

By fall 2021, a third of families with low-income and with children under five were unable to secure child care.[13] Many caregivers – mostly women –had to reduce their work hours or leave the workforce entirely.[16, 17] This conflict is reflected in nationwide employment trends: half of families with low-income and families of color had a household member lose a job or work hours within the first month of the pandemic.[14] For many families, these disruptions worsened pre-existing financial insecurity, challenging their ability to afford rent or mortgage payments.[7, 8, 15] Pandemic-related housing insecurity also increased, possibly resulting in higher rates of displacement.[18]

Simultaneously, signals of adverse health across multiple domains – including general health, mental health, and financial health – became more prevalent, particularly among racially and economically marginalized groups.[9, 19–21] Research taking place before and throughout the pandemic suggest that employment and housing disruptions may be partially responsible, as they can increase economic deprivation, destabilize established social networks and medical care, and subsequently induce a sequelae of interrelated adverse health effects that worsen mental and general health, as well as individuals’ ability to achieve food security.[9, 22–32] A limited but growing body of evidence also suggests that child care disruptions contribute to poor mental health and food insecurity,[9, 29] possibly by way of causing employment or housing disruptions, inducing personal and familial stress, increasing isolation, and reducing sleep. Independently and in tandem, indicators of these adverse health states – including depressive symptoms, low self-rated health, and self-reported food insecurity – have been associated with poor short and long-term outcomes among caregivers and their children, including cardiovascular disease, psychological impairments, and all-cause mortality.[27, 33–38]

Understanding the influence of pandemic-related social and economic disruptions on key indicators of wellbeing is critical for establishing policy priorities that can inform future public health emergency preparedness strategies and mitigate further harm. Yet, there is limited research assessing their comparative impacts on different health domains. We draw upon data from the Assessing Communities’ Experiences with Safety Net Supports Survey (ACCESS) to better understand the extent to which employment, housing, and child care disruptions during the COVID-19 pandemic were associated with depressive symptoms, self-rated health, and food security status among a racially and ethnically diverse sample of female, economically disadvantaged caregivers of young children in California.

## Methods

### Study setting

California is the second-most diverse state and home to over 39 million people (nearly 12% of the US population).[39, 40] After accounting for basic expenses and government benefits, it has the second highest poverty rate (15.4%) in the country; millions of residents participate in state and federal safety net programs. [41–44] At the onset of the pandemic, the state rolled out numerous social programs expansions (e.g., Special Supplemental Nutrition Program for Women, Infants and Children (WIC), the Supplemental Nutrition Assistance Program (SNAP/CalFresh), Unemployment Insurance (UI), and Temporary Assistance for Needy Families (TANF/CalWorks)) as well as newly created programs (e.g., Pandemic Electronic Benefit Transfer (P-EBT), Economic Impact Payments (federal stimulus checks), and eviction moratoria) to provide a more robust support network for vulnerable families.[45] The overlap between the implementation of these programs and our study period offered us a unique opportunity to explore the interplay between socioeconomic disruptions and health amidst the shifting social policy landscape.[45]

### Recruitment and study sample

ACCESS study staff recruited a racially and ethnically diverse convenience sample of caregivers who met the following criteria: (1) lived in California at the time of the interview, (2) had at least one dependent under 9 years of age, and (3) were likely eligible for the Earned Income Tax Credit (EITC) based on self-reported income and other demographic characteristics (the ACCESS study was initially designed to examine barriers to EITC take-up, although study goals shifted due to the COVID-19 pandemic). Potential participants were recruited in partnership with community-based organizations including safety net programs, social services agencies, tax preparation services, and other local organizations. ACCESS study staff also used snowball sampling as a recruitment strategy, asking those who completed the survey to share study information with eligible individuals within their networks.

**Fig. 1:**
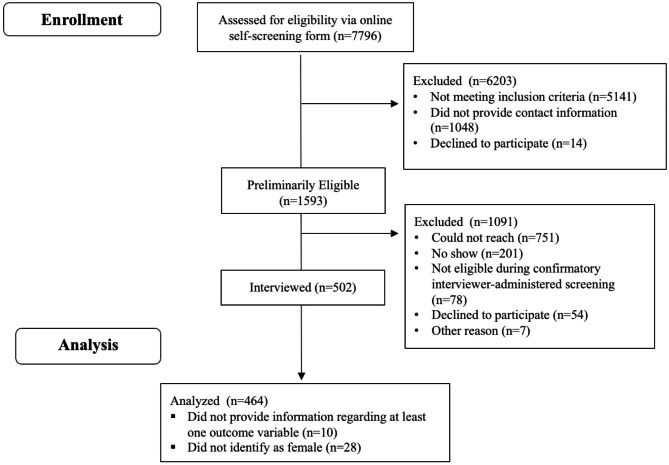
Sample selection diagram

Potential participants were asked to respond to an online eligibility screening questionnaire, which collected sociodemographic information and contact information, as well as consent to participate in the study. Individuals who responded to the screening questionnaire (n = 7,796) and were deemed eligible to participate (n = 1,593) were contacted by the study team via text message or telephone to schedule an interview. About 32% (n = 502) of eligible individuals were ultimately interviewed (see **Fig. **1**Sample Selection Diagram**). Additional study details are described in prior work.[46] We restricted this analysis to study participants who provided information regarding at least one outcome of interest and identified as female (n = 464).

### Study procedures

Survey-based interviews were conducted in English or Spanish via password-protected video conferencing software or by telephone from August 2020 – May 2021. Interviews lasted 1-1.5 hours. Interviewers were trained to handle sensitive topics such as finances, family matters, discrimination, and mental health; when possible, their racial and ethnic identities were concordant with interviewees. Responses were recorded using Qualtrics software. Participants received a $35-$50 honorarium based on interview completion. All study protocols were approved by the Committee for Protection of Human Subjects at the University of California, Berkeley.

### Survey development

Survey development was informed by a review of the literature, input from the study’s Community Advisory Board, and expert review. Previously validated items and scales were used whenever possible, as were validated translations. The survey instrument was pilot-tested in English (n = 12) and Spanish (n = 4), with alterations made to reduce completion time and improve clarity. The instrument included personal and household sociodemographic characteristics, including safety net program participation; experiences with employment, child care, and housing during the COVID-19 pandemic; and indicators of several health domains.

### Exposures

We created three binary exposure variables to examine disruptions participants experienced related to employment, child care, and housing since March 2020. If a participant responded affirmatively to at least one binary question within a given domain, they were deemed to have experienced that disruption.

To classify experiences of employment disruption, we asked participants whether they or their partner had reduced their work hours or lost a job. For child care, we asked whether child care or babysitting were unavailable when needed, or whether participants had difficulty taking care of children at home. Lastly, to determine housing, we asked participants whether they moved because they were having difficulty paying their rent or mortgage, afraid they would be evicted, or because they were evicted.

### Outcomes

We examined three health outcomes that were likely to be affected by the above disruptions: (1) depressive symptoms, (2) self-rated health, and (3) food security status.

Respondents’ depressive symptoms over the past week were measured using the validated, 10-item Center for Epidemiological Studies Depression Scale (CESD-10).[47] While originally developed for older adults, the CESD-10 has been validated among working age adults as well.[48, 49] Scores range from 0 to 30, with higher scores indicating more severe depressive symptoms. Using standard protocol, participants with scores ≥ 10 were classified as experiencing clinically meaningful depressive symptoms.[47]

Respondents’ self-rated health since the onset of pandemic was measured using a validated Likert scale item: “Since March 2020, would you say your health in general has been excellent, very good, good, fair, or poor?” This health measure has been consistently associated with markers of morbidity and all-cause mortality across diverse populations and time.[36, 37] We dichotomized this variable into low (fair/poor) vs. high (good/very good/excellent health) self-rated health, as is frequently seen in the literature.[21, 26, 50]

Respondents’ food security status from the past thirty days was measured using the validated, six-item short-form of the US Department of Agriculture’s (USDA) Household Food Security Survey Module.[51] Questions correspond to concerns or reductions of food quantity or quality within the context of affordability.[52] In accordance with the USDA scoring guidelines, we classified participants endorsing one or fewer questions as food-secure, and those endorsing two or more as food insecure.

### Covariates

We identified covariates that may confound the relationships between our exposures and outcomes: demographics (race/ethnicity, age, marital status, US vs. foreign born), indicators of socioeconomic status (education, pre-COVID-19 employment, pre-COVID-19 income, home ownership), household composition (number of children, number of adults, and whether caring for an infant), and timing of participants’ interviews (August 2020 – January 2021 vs. February – May 2021). We did not want to pose any potential or perceived risks to participants by asking them about their citizenship status; while foreign-born is an imperfect proxy, it is commonly employed by research agencies, including the US Census, to describe a related demographic characteristic of a population that may correspond to relevant research questions, including safety net program eligibility.[53] We dichotomized the timing of interviews to reflect the pre/post periods for the 2020–2021 winter surge of COVID-19 infections; smaller intervals would result in underpowered analyses.

### Missing data

Four covariates and two exposure variables were missing one or two observations each. We conducted linear, logistic, and multinomial regressions to impute missing continuous, binary, and categorical values, respectively.[54] Each imputation model incorporated the exposure, outcomes, covariates, and other variables that could predict values for the unobserved data points. Missing values for outcome variables were rare (< 1%), and these were not imputed as this is thought to add noise to resulting estimates.[55]

### Statistical analysis

We first calculated univariate statistics to describe the distribution of covariates, exposures, and outcomes. Next, we used chi-square and Wilcoxon rank sum tests to test for statistically significant (p < 0.05) differences in the distribution of covariates and outcomes by each exposure (e.g., whether someone experienced a child care disruption).

We then conducted a series of multivariable Poisson regressions with robust standard errors [56] to estimate prevalence ratios, adjusting for all covariates. We ran nine models that assessed the association of each of the three disruptions with each of the three outcomes (individual models), and an additional three models (one for each outcome) that included all exposures in the same regression to determine their respective influence on each outcome (referred to below as joint models).

### Secondary analyses

We hypothesized that employment disruptions may be more impactful among households who were mostly employed leading to the pandemic. To assess this, we ran three additional individual models and three joint models restricted to participants who reported that they or their partner were employed for most of 2019 (N = 374).

To assess whether the impacts of each disruption varied throughout the study period, we ran additional individual and joint models that included a binary interaction term between each exposure variable and the interview date (before vs. on or after February 1, 2021). Ward tests were used to gauge whether the interaction term was statistically significant (p < 0.05).

Safety net program participation had the potential to impact the relationships between our exposure and outcome variables. Given concerns about temporality, collinearity, and insufficient statistical power, we descriptively assessed participation rates among participants who experienced each disruption using bivariate analyses (including employment disruptions in the restricted sample). The safety net programs we examined included nutritional assistance programs (WIC, SNAP/CalFresh, and P-EBT), as well as cash-based assistance programs (UI, TANF/CalWorks, and federal stimulus checks). We lacked sufficient data to examine the relationships between disruptions and uptake of housing assistance programs.

Finally, we calculated Romano-Wolf stepdown p-values [57] to correct for multiple hypothesis testing while accounting for dependency between multiple exposures and outcomes within each set of analyses (main effects models, restricted employment models, interaction models).

All analyses were conducted using Stata 14.0 (College Station, TX).

## Results

Most participants were ages 25–34 years, female, Latinx, employed for the majority of 2019 (or had a partner that was employed), and attended some college or more (Table 1**)**. The median household income in 2019 was $18,900 (IQR: $9,200 − $30,900). There were several differences in distribution of sociodemographic characteristics by outcome status. Pandemic-related child care (70.0%) and employment (63.4%) disruptions were common, but only 7.5% of participants reported experiencing housing disruptions. Nearly 44.2% of respondents reported experiencing depressive symptoms over the past week, 33.2% reported low self-rated health since the onset of the pandemic, and 35.9% reported food insecurity over the past thirty days. Disruptions to child care and housing were both positively associated with each adverse outcome.

**Table 1:** Sample sociodemographic characteristics, stratified by pandemic-related employment, child care, and housing disruptions

	Total (N = 646)	Depressive Symptoms (N = 205)		Low Self-Rated Health (N = 154)		Food Insecurity (N = 166)	
*N (%); Median (IQR)*							
**Race/ethnicity**							
Latinx/Hispanic	272	107 (39.3%)		103 (37.9%)	*	104 (38.2%)	**
Non-Hispanic Black	96	45 (46.9%)		19 (19.8%)	*	28 (29.5%)	**
Non-Hispanic White	46	30 (65.2%)		10 (21.7%)	*	19 (41.3%)	**
Other	50	23 (46.0%)		22 (44.0%)	*	15 (30.0%)	**
**Age (years)**							
18–24	60	21 (35.0%)		15 (25.0%)		25 (41.7%)	
25–34	250	116 (46.4%)		86 (34.4%)		84 (33.6%)	
35+	154	68 (44.2%)		53 (34.4%)		57 (37.3%)	
**Married/partnered**	197	83 (42.1%)		71 (36.0%)		66 (33.5%)	
**Foreign born**	347	39 (33.3%)	**	38 (32.5%)		39 (33.3%)	
**Education**							
High school or less	141	54 (38.3%)	*	44 (31.2%)		62 (44.0%)	*
Some college	233	116 (49.8%)	*	77 (33.0%)		84 (36.2%)	*
Bachelors or greater	90	35 (38.9%)	*	33 (36.7%)		20 (22.2%)	*
**2019 employment**							
1 + partner mostly full-time	300	127 (42.3%)		98 (32.7%)		108 (36.1%)	
1 + partner mostly part-time	74	31 (41.9%)		27 (36.5%)		21 (28.4%)	
Mostly unemployed	89	47 (52.8%)		29 (32.6%)		37 (41.6%)	
**Home owner**	84	33 (39.3%)		32 (38.1%)		20 (23.8%)	*
**Adults/household**	2.0 (1.0–2.0)	2.0 (1.0–2.0)		2.0 (2.0–3.0)		2.0 (1.0–2.0)	
**Children/household**	2.0 (1.0–3.0)	2.0 (1.0–3.0)		2.0 (1.0–3.0)		2.0 (1.0–3.0)	
**Caring for an infant**	291	133 (45.7)		104 (35.7%)		109 (37.6%)	
**2019 income, in thousands USD**	18.9 (9.2–30.9)	18.9 (9.5–29.4)		20.5 (11.0-31.1)		18.5 (8.2–29.0)	
**Interview** ≥ **Feb 1, 2021**	259	109 (42.1%)		84 (32.4%)		75 (29.1%)	**
**Disruption**							
Child care	325	167 (51.4%)	***	119 (36.6%)	*	129 (39.8%)	**
Employment	294	132 (44.9%)		105 (35.71)		114 (38.9%)	
Housing	35	23 (65.7%)	**	17 (48.6%)	*	24 (68.6%)	***

Sample was drawn from the ACCESS Study. Row percentages shown. Scores ≥ 10 on the Center for Epidemiologic Studies Depression Scale were classified as experiencing clinically meaningful depressive symptoms. Self-rated health was dichotomized into fair/poor vs. good/very good/excellent health. Food insecurity was determined by a score of ≥ 2 on the US Department of Agriculture’s 6-item Household Food Security Survey Module

Feb = February

*p < 0.05; **p < 0.01; ***p < 0.001

In the adjusted main effects models, child care disruptions were associated with depressive symptoms (PR: 1.86, 95% CI: 1.38, 2.49), lower self-rated health (PR: 1.54, 95% CI: 1.10, 2.16) and food insecurity (PR: 1.53, 95% CI: 1.12, 2.09) (Table 2). These associations remained when controlling for employment and housing disruptions in joint models. Housing disruptions were also associated with depressive symptoms (PR: 1.52, 95% CI: 1.20, 1.93), lower self-rated health (PR: 1.89, 95% CI: 1.33, 2.68) and food insecurity (PR: 1.81, 95% CI: 1.39, 2.37). These associations also remained statistically significant when controlling for the other disruptions. Most of these findings were robust to Romano-Wolf p-value adjustments (Table 2). In contrast, employment disruption was not associated with any outcome in the full sample or restricted sample of families that worked for most of 2019 (Table 2).

**Table 2:** Pandemic-related socioeconomic disruptions and female caregivers’ health

	Depressive Symptoms	Low Self-Rated Health	Food Insecurity
**Disruption Experienced**	PR (95% CI)	PR (95% CI)	PR (95% CI)
***Individual Models***			
Child care	1.86^**‡^ (1.38, 2.49)	1.54^*†^ (1.10, 2.16)	1.53^**†^ (1.12, 2.09)
Employment – full sample	1.07 (0.86, 1.33)	1.24 (0.94, 1.63)	1.26 (0.96, 1.65)
Employment – restricted sample	1.15 (0.88, 1.50)	1.15 (0.85, 1.55)	1.39 (< 1.00, 1.93)
Housing	1.52^**‡^ (1.20, 1.93)	1.89^**‡^ (1.33, 2.68)	1.81^**‡^ (1.39, 2.37)
***Joint Models***			
Child care	1.80^**‡^ (1.34, 2.42)	1.47^*^ (1.05, 2.06)	1.42^*^ (1.04, 1.95)
Employment – full sample	1.03 (0.84, 1.28)	1.20 (0.91, 1.57)	1.20 (0.92, 1.57)
Employment – restricted sample	1.12 (0.87, 1.45)	1.11 (0.82, 1.50)	1.34 (0.97, 1.86)
Housing	1.36^*^ (1.06, 1.74)	1.74^*‡^ (1.23, 2.48)	1.65^**‡^ (1.25, 2.18)

Full (N = 464) and restricted (N = 374) samples were drawn from the ACCESS Study. Restricted sample excluded participants whose families were not employed (full- or part-time) for most of 2019. Results were derived from multivariable Poisson models with robust standard errors; each adjusted for race/ethnicity, age, marital status, foreign-born status, educational attainment, pre-COVID-19 employment, pre-COVID-19 income, home ownership, number of children, number of adults, caring for an infant, and timing of participants’ interviews. Individual models include just one disruption as the exposure variable; joint models contain all three

Scores ≥ 10 on the Center for Epidemiologic Studies Depression Scale were classified as experiencing clinically meaningful depressive symptoms. Self-rated health was dichotomized into fair/poor vs. good/very good/excellent health. Food insecurity was determined by a score of ≥ 2 on the US Department of Agriculture’s (USDA) 6-item Household Food Security Survey Module

* p < 0.05, ** p < 0.01, ***p < 0.001, † Romano-Wolf adjusted p < 0.10, **‡** Romano-Wolf adjusted p < 0.05

The relationships between housing and depressive symptoms (individual and joint models) as well as employment and low self-rated health (individual and joint models) were modified by the timing of participants’ interviews (Table 3). However, none of these associations were robust to Romano-Wolf p-value adjustments.

**Table 3:** Pandemic-related socioeconomic disruptions and caregivers’ health, with effect modification by participants’ interview date

	Depressive Symptoms	Wald	Low Self-Rated Health	Wald	Food Insecurity	Wald
**Disruption Experienced**	PR (95% CI)		PR (95% CI)		PR (95% CI)	
***Individual Models***						
**Employment**		0.9452		0.0381		0.4442
<Feb 1, 2021^ A^	1.06 (0.77, 1.46)		1.79^*^ (1.10, 2.92)		1.12 (0.79, 1.58)	
≥Feb 1, 2021^ A^	1.00 (0.72, 1.39)		1.41 (0.86, 2.30)		0.84 (0.57, 1.23)	
≥Feb 1, 2021^B^	0.96 (0.75, 1.23)		0.92 (0.68, 1.25)		0.78 (0.59, 1.03)	
**Child Care**		0.7837		0.9183		0.3367
<Feb 1, 2021^ A^	1.78^**^ (1.18, 2.69)		1.52 (0.96, 2.41)		1.34 (0.91, 1.96)	
≥Feb 1, 2021^ A^	1.68^*^ (1.12, 2.53)		1.42 (0.90, 2.23)		1.00 (0.67, 1.47)	
≥Feb 1, 2021^B^	1.09 (0.88, 1.36)		1.05 (0.80, 1.38)		0.81 (0.62, 1.05)	
**Housing**		0.0194		0.2907		0.5017
<Feb 1, 2021^ A^	1.16 (0.82, 1.64)		2.21^**^ (1.50, 3.26)		1.66^**^ (1.23, 2.24)	
≥Feb 1, 2021^ A^	1.83^**^ (1.32, 2.53)		1.48 (0.77, 2.84)		1.36 (0.88, 2.10)	
≥Feb 1, 2021^B^	1.80^**^ (1.31, 2.48)		1.34 (0.70, 2.56)		1.77 (0.83, 1.94)	
***Joint Models***						
**Employment**		0.9009		0.0261		0.4914
<Feb 1, 2021^ A^	1.02 (0.75, 1.38)		1.76* (1.10, 2.81)		1.08 (0.77, 1.52)	
≥Feb 1, 2021^ A^	0.96 (0.70, 1.32)		1.36 (0.85, 2.20)		0.81 (0.56, 1.17)	
≥Feb 1, 2021^B^	0.95 (0.75, 1.21)		0.91 (0.67, 1.22)		0.77 (0.58, 1.01)	
**Child Care**		0.7343		0.8797		0.2913
<Feb 1, 2021^ A^	1.71^*^ (1.13, 2.60)		1.44 (0.91, 2.26)		1.23 (0.83, 1.82)	
≥Feb 1, 2021^ A^	1.64^*^ (1.09, 2.47)		1.38 (0.88, 2.16)		0.94 (0.63, 1.39)	
≥Feb 1, 2021^B^	1.10 (0.89, 1.37)		1.06 (0.81, 1.39)		0.81 (0.62, 1.05)	
**Housing**		0.0154		0.2819		0.5090
<Feb 1, 2021^ A^	1.02 (0.73, 1.42)		2.05^**^ (1.38, 3.03)		1.52^**^ (1.13, 2.06)	
≥Feb 1, 2021^ A^	1.63^**^ (1.15, 2.33)		1.36 (0.71, 2.60)		1.25 (0.80, 1.95)	
≥Feb 1, 2021^B^	1.63^**^ (1.15, 2.31)		1.23 (0.65, 2.34)		1.17 (0.76, 1.82)	

Sample was drawn from the ACCESS Study (N = 464). Results were derived from multivariable Poisson models with robust standard errors; each assessed for effect modification by timing of participants’ interviews and adjusted for race/ethnicity, age, marital status, foreign-born status, educational attainment, pre-COVID-19 employment, pre-COVID-19 income, home ownership, number of children, number of adults, and caring for an infant

Scores ≥ 10 on the Center for Epidemiologic Studies Depression Scale were classified as experiencing clinically meaningful depressive symptoms. Self-rated health was dichotomized into fair/poor vs. good/very good/excellent health. Food insecurity was determined by a score of ≥ 2 on the US Department of Agriculture’s 6-item Household Food Security Survey Module

Feb = February

^A^ The reference group did not experience disruption and was interviewed before February 1, 2021

^B^ The reference group did not experience disruption and was interviewed on or after February 1, 2021

^*^p < 0.05, ^**^p < 0.01, ***p < 0.001, ^†^Romano-Wolf adjusted p < 0.10, ^**‡**^Romano-Wolf adjusted p < 0.05

### Pandemic safety net program participation by disruption status

Food-based safety net participation was high among all participants, although there was a higher prevalence of SNAP participation among individuals who experienced child care and housing disruptions; this was also true for participation in TANF (**Appendix A**). Participants who reported employment disruptions reported higher rates of UI participation. There were no meaningful differences between the full sample and restricted sample of individuals who worked for most of 2019.

## Discussion

We assessed the extent to which three pandemic-related socioeconomic disruptions – employment, child care, and housing – were associated with depressive symptoms, self-rated health, and food security among a diverse sample of female caregivers with low income and young children. Most notably, we found that child care disruptions were strongly associated with depressive symptoms, and to a lesser extent, low self-rated health and food insecurity. The risk of depressive symptoms among caregivers reporting child care disruptions even exceeded that of caregivers reporting major housing disruptions.

The prevalence and severity of child care disruptions suggest that pandemic-related assistance was an insufficient substitute for reliable infrastructure within this sample. Although prior research has demonstrated that school and daycare closures have dramatically reduced caregivers’ workforce participation and subsequently increased economic insecurity, the null effects of our employment models indicate that other mediators may have been driving the associations we observed between child care disruption and adverse health outcomes .[14, 17] It is possible that factors for which there were no tangible supports – such as heightened parental burnout (resulting from upended schedules and routines, additional supervisory and educational roles, and reduced external social supports) – may have manifested in greater depressive symptoms and worse self-reported health. [58–61] Gaps in existing programming may have similarly contributed. For example, reduced availability of school and child care-based meals may have elevated the risk of food insecurity in caregivers’ households, (further) contributing to adverse general and mental health outcomes [27, 62, 63]. Even though alternative nutritional assistance programs were made available to families with eligible school-aged children, these benefits were not as easily accessible for families with young children missing meals otherwise provided in child care settings outside of the public school system.[45, 62].

Similar to other studies, [24–26] we also found that caregivers reporting housing disruptions had markedly greater health risks, although the proportion of our sample that experienced them was still small. These findings may also elucidate strengths and weaknesses of pandemic safety net policies. On one hand, the rarity of housing disruption within this low-income sample contributes to research suggesting that the COVID-19 eviction moratoria and household-level financial assistance may have helped to prevent more widespread displacement.[45, 64] However, housing disruptions persisted despite these efforts, indicating that protection from such supports was not universally accessed or sufficient. That housing disruption and depressive symptoms appeared to be modified by time further suggests that they may lose their potency as supports may wane. [45]

While the prevalence of employment disruptions was high, our study did not document meaningful associations between employment disruptions and depressive symptoms, self-rated health, or food security status. One potential explanation for the limited influence of employment disruption could be that job and/or wage loss were ameliorated by subsequent unemployment or additional cash-based safety net supports.[45, 65] Safety net program expansions during COVID-19 included a combination of state and federal relief dollars that were used to increase the length and dollar amount of UI, provide stimulus payments of up to $1,400 per person, and bolster the dollar amount and availability of food assistance for families with children.[45] Nearly half of the participants who reported employment disruption received UI, which provided between $340 - $1,050 to recipients each week through March 2021 (notably, the maximum amount exceeded many participants’ 2019 income). An even larger percentage of participants received economic stimulus payments and food assistance benefits, which could have supplemented lost income for participants whether or not they received UI. Parents of young children who lost employment but had access to adequate financial support may have therefore incurred fewer social needs and related stress, as has previously been observed.[65].

This study has several strengths. It is among the first to assess the potential health repercussions of child care disruptions relative to other pandemic-related disruptions. Further, describing the magnitude of employment, child care, and housing disruptions among a diverse group of economically marginalized caregivers, most of whom were connected to at least one federal food or financial assistance program, broadens our understanding of the potential impacts and elucidates possible shortfalls of the social safety net among the individuals they served during a public health emergency.

Our findings should also be interpreted in light of their limitations. Using different timescales for each of our outcome measures may have introduced measurement bias. Similarly, using a cross-sectional study design with overlapping exposure and outcome periods precluded us from making causal inferences and ruled out our ability to conduct mediation analyses. We also recruited a convenience sample that did not represent the California or US population, limiting the generalizability of our results and potentially introducing selection bias. For example, our sample did not adequately represent individuals of Asian or Pacific Islander descent (6% of our sample, which was grouped into the “other race/ethnicity” category vs. 16% of Californians), overrepresented Black and Latinx individuals (21% and 59% of our sample vs. 7% and 40% of Californians, respectively) and excluded male participants altogether.[40] Nevertheless, the richness of the data provide additional insight that is not possible with larger state or national surveys. Additionally, we may have been underpowered for some analyses, particularly housing disruption models given that this exposure was less common, as well as effect modification given the small cell sizes. Our relatively small sample and limited statistical power also precluded us from being able to meaningfully examine the severity of our exposure variables, which, in turn, led us to aggregate related but distinct experiences (e.g., job loss and wage loss).

We could not account for variations in the severity or timing of disruptions between participants, nor unmeasured confounders such as citizenship status, receipt of formal or informal social supports, or financial insecurity prior to the pandemic. Our results are also prone to self-reporting and recall biases; we tried to minimize these issues by using standardized instruments and protocols whenever possible. Finally, while our goal was to conduct a within-group analysis, this prevented us from exploring differential impacts of pandemic-related stressors among distinct populations. Future research should explore how disruptions affected health among more advantaged populations with additional resources to cope with these stressors.

## Conclusion

This study broadens our understanding of the association between socioeconomic disruptions and several indicators of health among female caregivers with low income and young children. We found that participants reporting child care and housing disruptions had a greater likelihood of experiencing depressive symptoms, worse self-rated health, and food insecurity than those who reported employment disruptions. This suggests that policies and other supports for those facing child care and housing disruptions during the pandemic were less adequate than policies around employment and cash assistance. While our findings may not correspond to disruptions that extend beyond the context of the pandemic and related mitigation policies, they offer a window into the vulnerabilities of the US safety net. Attending to structural deficits in the child care system and increasing the availability of affordable housing is critical for supporting the health of families with young children.

## Electronic supplementary material

Below is the link to the electronic supplementary material.

Supplementary Material 1

## Data Availability

Data that support the findings of this study are available upon reasonable request from the corresponding author (EMB). They are not publicly available due to the sensitivity of information collected from participants.

## List of abbreviations

US: United States.
ACCESS: Assessing California Communities’ Experiences with Safety Net Supports Survey.
EITC: Earned Income Tax Credit.
USDA: United States Department of Agriculture.
WIC: Special Supplemental Nutrition Program for Women, Infants and Children.
SNAP: Supplemental Nutrition Assistance Program.
P-EBT: Pandemic Electronic Benefit Transfer.
UI: Unemployment Insurance.
TANF: Temporary Assistance for Needy Families.
PR: Prevalence Ratio.
CI: Confidence Interval.
