# Supplementary material for: Pandemic-related socioeconomic disruptions and adverse health outcomes: a cross-sectional study of female caregivers
Source: BMC Public Health. 2022 Oct 11;22:1893. doi: 10.1186/s12889-022-14287-2 (PMC9552726; doi:10.1186/s12889-022-14287-2)
Supplement: Supplementary file 1 — Supplementary Material 1 [file 12889_2022_14287_MOESM1_ESM.docx]

**Appendix A**. Differences in safety net program participation rates during the pandemic

|  | **Total**  **(N=464)** | **Experienced employment disruption and worked for most of 2019 (N=266)** | **Experienced Employment Disruption (N=294)** | **Experienced**  **Child Care Disruption (N=325)** | **Experienced Housing**  **Disruption (N=35)** |
| --- | --- | --- | --- | --- | --- |
|  | N (%) | | | | |
| **Federal food assistance** | 444 (96%) | 253 (95.8%) | 281 (96.2%) | 312 (96.3%) | 35 (100.0%) |
| WIC | 363 (78.6%) | 205 (77.7%) | 227 (77.7%) | 250 (77.2%) | 26 (74.3%) |
| SNAP | 265 (57.5%) | 147 (55.9%) | 169 (58.1%) | 197 (61.0%)* | 26 (74.3%)* |
| P-EBT | 236 (51.1%) | 141 (53.4%) | 152 (52.1%) | 172 (53.1%) | 18 (51.4%) |
| **Cash assistance** |  |  |  |  |  |
| UI | 177 (38.3%) | 126 (47.7%)* | 137 (46.9%)* | 124 (38.3%) | 13 (37.1%) |
| Federal stimulus | 397 (85.9%) | 226 (85.6%) | 246 (84.2%) | 279 (86.1%) | 27 (77.1%) |
| TANF | 123 (26.6%) | 67 (25.4%) | 80 (27.4%) | 98 (30.2%)* | 17 (48.6%)* |

Sample was drawn from the ACCESS Study (N=492). Column percentages shown.

WIC = Special Supplemental Nutrition Program for Women, Infants and Children

SNAP = Supplemental Nutrition Assistance Program

P-EBT= Pandemic Electronic Benefit Transfer

Federal stimulus = Economic Impact Payment

UI = Unemployment Insurance

TANF = Temporary Assistance for Needy Families

* p<0.05 difference between distribution of participants who reported experiencing the stated disruption and those that did not
